# Angiotensin Converting Enzyme Inhibitors and the Reduced Risk of Alzheimer’s Disease in the Absence of Apolipoprotein E4 Allele

**DOI:** 10.3233/JAD-130716

**Published:** 2013

**Authors:** Wei Qiao Qiu, Mkaya Mwamburi, Lilah M. Besser, Haihao Zhu, Huajie Li, Max Wallack, Leslie Phillips, Liyan Qiao, Andrew E. Budson, Robert Stern, Neil Kowall

**Affiliations:** a Department of Pharmacology & Experimental Therapeutics, Boston University School of Medicine, Boston, MA, USA; b Department of Psychiatry, and Boston University School of Medicine, Boston, MA, USA; c Alzheimer’s Disease Center, Boston University School of Medicine, Boston, MA, USA; d Department of Public Health and Family Medicine, Tufts University, Boston, MA, USA; e National Alzheimer’s Coordinating Center, University of Washington, Seattle, WA, USA; f Neurology Department, the First People’s Hospital of Changzhou, Changzhou, China; g Urban Indian Health Institute, Seattle WA, USA; h Qinghua University Yuquan Hospital, Beijing, China

**Keywords:** Alzheimer’s disease, apolipoprotein E4 allele (ApoE4), angiotensin converting enzyme (ACE) inhibitor

## Abstract

Our cross-sectional study showed that the interaction between apolipoprotein E4 (ApoE4) and angiotensin converting enzyme (ACE) inhibitors was associated with Alzheimer’s disease (AD). The aim of this longitudinal study was to differentiate whether ACE inhibitors accelerate or reduce the risk of AD in the context of ApoE alleles. Using the longitudinal data from the National Alzheimer’s Coordinating Center (NACC) with ApoE genotyping and documentation of ACE inhibitors use, we found that in the absence of ApoE4, subjects who had been taking central ACE inhibitor use (χ^2^ test: 21% versus 27%, *p* = 0.0002) or peripheral ACE inhibitor use (χ^2^ test: 13% versus 27%, *p* < 0.0001) had lower incidence of AD compared with those who had not been taking an ACE inhibitor. In contrast, in the presence of ApoE4, there was no such association between ACE inhibitor use and the risk of AD. After adjusting for the confounders, central ACE inhibitor use (OR = 0.68, 95% CI = 0.55, 0.83, *p* = 0.0002) or peripheral ACE inhibitor use (OR = 0.33, 95% CI = 0.33, 0.68, *p* < 0.0001) still remained inversely associated with a risk of developing AD in ApoE4 non-carriers. In conclusion, ACE inhibitors, especially peripherally acting ones, were associated with a reduced risk of AD in the absence of ApoE4, but had no such effect in those carrying the ApoE4 allele. A double-blind clinical trial should be considered to determine the effect of ACE inhibitors on prevention of AD in the context of ApoE genotype.

## INTRODUCTION

Angiotensin converting enzyme (ACE) inhibitors are effective hypertension medications and are commonly used in the elderly [1, 2]. The relationship between ACE inhibitor use and the risk of Alzheimer’s disease (AD) is unclear, with conflicting results reported in the literature [3, 4]. One study found that peripheral ACE inhibitors are associated with an increased risk of AD [5], while others indicated that peripheral ACE inhibitors reduce dementia risk [6, 7]. Our recent cross-sectional study found that ACE inhibitor use was positively associated with AD only among apolipoprotein E4 carriers (ApoE4), but not among ApoE4 non-carriers [8]. There were two possibilities: 1) ACE inhibitors accelerate the development of AD in the presence of ApoE4 or 2) ACE inhibitors delay the onset of AD in ApoE4 non-carriers. As the relationship between ACE inhibitors and the development of AD in the context of ApoE alleles is unclear, we conducted a longitudinal study to clarify these two possibilities.

The ApoE4 allele is the major genetic risk factor of late-onset and sporadic AD [9] and memory decline [10] as well as vascular diseases. However, 50% of AD patients do not have the ApoE4 allele and not all ApoE4 carriers develop AD, even at very old age [11]. Thus there are probably other factors interacting with ApoE alleles to either accelerate or delay the development of AD. Many clinical trials, especially in oncology, demonstrate the importance of personalized medicine by showing that different genetic profiles respond to certain chemotherapies differentially [12]. Since ApoE genotypes are associated with cerebrovascular pathology and the clearance of a major determinant of AD, amyloid-β peptide (Aβ), we hypothesized that ApoE alleles may interact with ACE inhibitors to influence AD development. As a follow-up to our previous cross-sectional study, we used the longitudinal data from the National Alzheimer’s Disease Coordinating Center (NACC) [13, 14] to determine whether ACE inhibitors are associated with a differential risk for the development of AD in ApoE4 carriers versus non-carriers. Because AD pathology is located in the brain, we also divided ACE inhibitors into central and peripheral ACE inhibitors based on whether they can pass through the blood-brain barrier.

## METHODS

### Study sample

NACC data collection was initiated in 1999 and funded by the National Institute on Aging (NIA) to develop and maintain a nation-wide database combining the data collected at the NIA-funded Alzheimer’s Disease Centers (ADCs) [13, 14]. Methods for the Uniform Data Set (UDS) collection have been previously published [14, 15]. This procedure was approved by the Institutional Review Board overseeing each ADC. All participants signed informed consents prior to participating in the NACC study. For this study analysis, 4,830 subjects from 33 ADCs in the longitudinal NACC study are included. These subjects were seen annually, starting in 2005, and this study included data collected through May 2011. We included only those subjects who had available ApoE genotype data, and for whom the use of ACE inhibitors was documented. We excluded those subjects who had dementia at baseline.

### Angiotensin converting enzyme inhibitors

Medication use was documented at each site and coded. For this study, ACE inhibitors at baseline were classified as one category [16]. Further, the ACE inhibitors including captopril, fosinopril, lisinopril, perindopril, rampril, and trandolapril were defined as central ACE inhibitors because they pass the blood-brain barrier. Peripheral ACE inhibitors (i.e., those not passing the blood-brain barrier) included benazepril, enalapril, moexipril, and quinapril.

### Diagnosis of Alzheimer’s disease

The diagnosis of dementia was based on DSM-IV criteria. NINCDS-ADRDA guidelines [17] were used to determine if diagnostic criteria were met for possible or probable AD. The conversion to AD dementia was defined by the new diagnosis of either probable or possible AD.

### Statistical analysis

Statistical analysis was performed using SAS (version 9.1). For analyses of baseline characteristics, the Chi-Square test (χ^2^ test) was used to compare proportions for binary and categorical variables. Continuous variables were presented as mean ± SD and compared using *T*-tests. We used each interval between annual visits as our analysis unit taking into account non-independence of study data due to repeated measures. To account for non-independence of repeated measures in the longitudinal analyses, generalized estimation equations (GEE) logistic regression with first order autoregression covariance matrix structure was used to examine associations between presence of AD at the end of the interval versus presence of ApoE4 or ACE inhibitor use while adjusting for age, gender, ethnicity, education, smoking, drinking and follow-up time. Baseline data on diabetes, hypertension, stroke, heart failure, amnestic MCI, and non-amnestic MCI were also used as covariates in the model. The interactions between ApoE4 and ACE inhibitor use were explored in the logistic regression models. For all analyses, the two-tailed alpha level of 0.05 was used.

## RESULTS

The analysis included 4,830 subjects who did not have dementia at baseline, and for whom information was available on ApoE genotype, ACE inhibitor use, and the follow-up diagnoses on AD. The average (mean ± SD) age was 76.5 ± 7.9 years old, and the average follow-up time was 3.4 ± 1.1 for this study sample. The majority was Caucasian (86%) and 48% were males. The average years of education were 15.1 ± 3.2. ApoE allele frequencies were ApoE2/2 or ApoE2/3 = 672/4830 (14%); ApoE3/3 = 2686/4830 (56%); ApoE3/4 or ApoE4/4 = 1342/4830 (28%), and ApoE2/4 = 150/4830 (3%). Thus, there were 1,492 subjects (31%) carrying at least one ApoE4 allele. The majority of subjects had hypertension (81%) and only a few had heart failure (6%); 3,255 subjects (67%) had been treated with an ACE inhibitor.

While there was no difference in taking central ACE inhibitors between those with and without an ApoE4 allele, slightly less ApoE4 non-carriers had been taking peripheral ACE inhibitors than ApoE4 carriers (12% versus 14%, *p* = 0.02) (Table 1). Medically, while there was no difference in the rate of hypertension between the two groups, slightly more ApoE4 non-carriers had diabetes (χ^2^ test: 19% versus 17%, *p* = 0.01), stroke (χ^2^ test: 6% versus 4%, *p* = 0.005), and heart failure (χ^2^ test: 7% versus 4%, *p* = 0.003) than ApoE4 carriers. ApoE4 non-carriers were older (mean ± SD: 77.3 ± 8.1 versus 74.6 ± 7.1, *p* < 0.0001), had longer follow-up time (mean ± SD: 3.5 ± 1.1 versus 3.3 ± 1.2, *p* < 0.0001), were more likely to report current smoking (χ^2^ test: 4% versus 3%, *p* = 0.01) and alcohol abuse (χ^2^ test: 5% versus 3%, *p* = 0.01) than ApoE4 carriers. While there were no differences in gender and education between those with and without an ApoE4 allele, more ApoE4 non-carriers were Caucasian than ApoE4 carriers (χ^2^ test: 87% versus 82%, *p* < 0.0001).

**Fig. 1:**
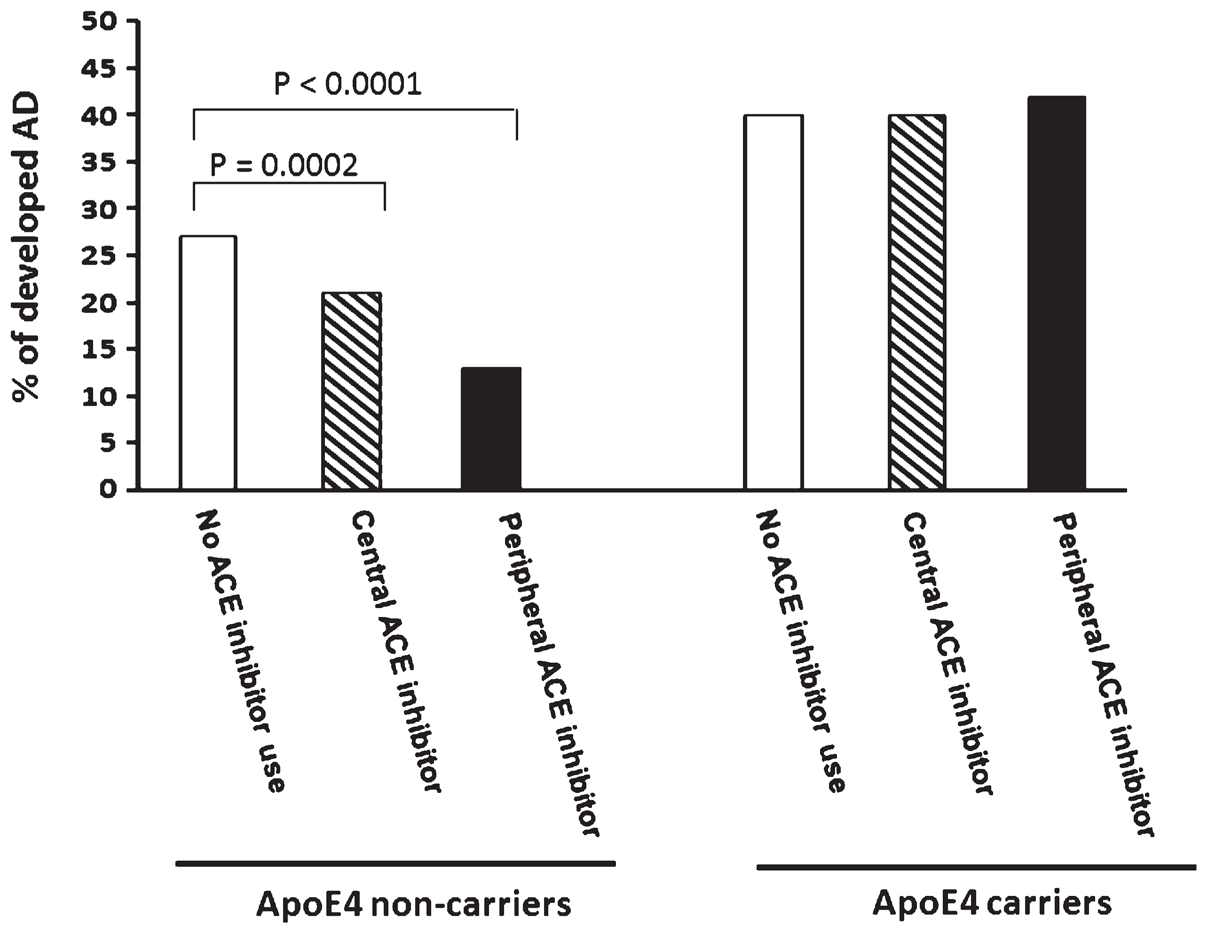
The onset of Alzheimer’s disease among those with and without the ACE treatment in the absence and presence of ApoE4 allele. The percentages of AD onset (combined probable AD and possible AD) were compared between different subgroups: in the absence of ApoE4 (ApoE4−) or presence of ApoE4 (ApoE4+) and further divided into no ACE inhibitor use, central ACE inhibitor use, and peripheral ACE inhibitor use. Chi square (χ^2^ test) was used to compare between the subgroup without ACE use and either ACE inhibitor subgroup. *p* values for the statistical significance between the two subgroups are shown.

As expected, ApoE4 carriers had an increased risk of developing probable (χ^2^ test: 30% versus 14%, *p* < 0.0001) or possible (χ^2^ test: 10% versus 8%, *p* = 0.01) AD compared with ApoE4 non-carriers (Table 1). We further divided both ApoE4 non-carriers and carriers into three subgroups based the usage of ACE inhibitor: 1) no ACE inhibitor use, 2) central ACE inhibitor use, or 3) peripheral ACE inhibitor use (Fig. 1). In the absence of ApoE4, both central ACE inhibitor use (χ^2^ test: 21% versus 27%, *p* < 0.0001) and peripheral ACE inhibitor use (χ^2^ test: 13% versus 27%, *p* = 0.0002) were associated with a further reduced risk of developing probable and possible AD, considered together, compared with those not taking ACE inhibitors. In contrast, ACE inhibitor use was not associated with the risk of developing AD dementia among ApoE4 carriers.

**Fig. 2:**
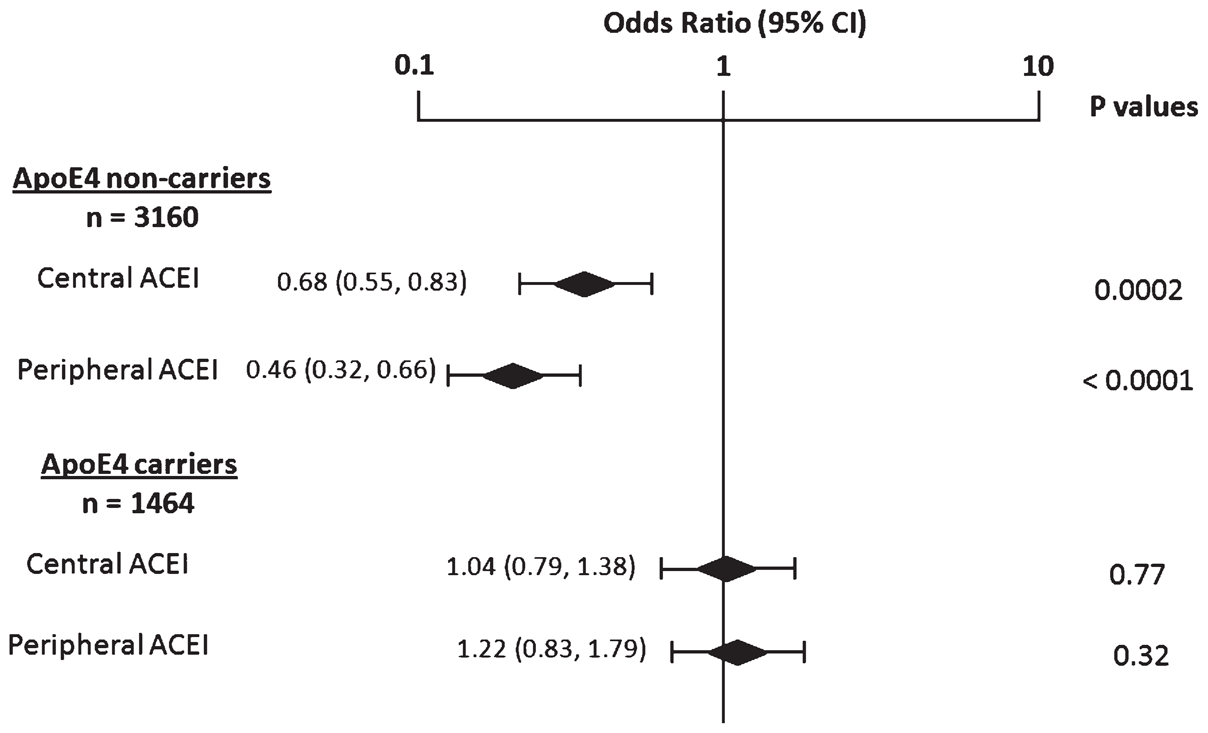
Effects of central versus peripheral ACE inhibitor use and the development of Alzheimer’s disease in ApoE4 non-carriers and ApoE4 carriers. The subjects were divided into those ApoE4 non-carriers and ApoE4 carriers. Using multivariate logistic regression models, we examined the associations between the central ACE inhibitor (central ACEI) versus peripheral ACE inhibitor (peripheral ACEI) and the development of AD after adjusting for the confounders in ApoE4 non-carriers or ApoE4 carriers separately. We used each interval between annual visits as our analysis unit taking into account non-independence of study data due to repeated measures. The confounders included age, gender, ethnicity, education, smoking, drinking, follow-up time, diabetes, hypertension, stroke, heart failure, amnestic MCI, and non-amnestic MCI. Odds ratios (95% CI) and *p* values are shown.

Results of multivariate logistic regression analysis (Table 2) indicate that central ACE inhibitor use (OR = 0.85, 95% CI = 0.74, 0.98, *p* = 0.03) or peripheral ACE inhibitor use (OR = 0.68, 95% CI = 0.54, 0.86, *p* = 0.001) was inversely associated with a risk of developing AD dementia after adjusting for ApoE4 and other confounders including age, gender, ethnicity, education, smoking, drinking, and the follow-up time (Model I). Adding the variables of vascular diseases including diabetes, hypertension, stroke, and heart failure in addition to amnestic MCI and non-amnestic MCI to this model did not affect the relationship between either central ACE inhibitor use (OR = 0.79, 95% CI = 0.67, 0.93, *p* = 0.004) or peripheral ACE inhibitor use (OR = 0.73, 95% CI = 0.57, 0.94, *p* = 0.02) and a risk of developing AD (Model II). Further, the interaction between ApoE4 carrier status and central ACE inhibitor use (OR = 0.44, 95% CI = 0.33, 0.60, *p* < 0.0001) and the interaction between ApoE4 status and peripheral ACE inhibitor use (OR = 0.27, 95% CI = 0.16, 0.44, *p* < 0.0001) were associated with decreased risk of AD (Model III). Multivariate logistic regression was applied to ApoE4 non-carriers (*n* = 3,160) or carriers (*n* = 1,464) separately to study the relationship between ACE inhibitors and the risk of developing AD in this genotype (Fig. 2). Again, both central ACE inhibitor use (OR = 0.68, 95% CI = 0.55, 0.83, *p* = 0.0002) and peripheral ACE inhibitor use (OR = 0.46, 95% CI = 0.32, 0.66, *p* < 0.0001) were inversely associated with the development of AD in the absence of ApoE4. In contrast, among ApoE4 carriers, neither type of ACE inhibitor use was found to be associated with AD risk. The majority of subjects were on the same ACE inhibitors from baseline to follow-ups. The conclusions remained the same after we added the variables of drug changes at each visit (data not shown).

## DISCUSSION

Because current estimates predict that there will be 13 million AD patients in the US by 2050 [18], development of prevention strategies and effective disease modification methods are critically important. Using a cross-sectional sample, we found that the interaction between ApoE4 and ACE inhibitor use was associated with AD [8], raising a possibility that ACE inhibitors may influence the development of AD based on ApoE4 genotype. To follow up this question, we used the longitudinal NACC data and found that ACE inhibitors were associated with lower incidence of AD in the absence of ApoE4, but there was no such association in the presence of ApoE4 (Table 2 and Fig. 2). The advantage in using the NACC data was that all the diagnoses of dementia were through NIH supported Alzheimer’s Disease Centers in the US. Our study suggests that ACE inhibitors may be beneficial and useful in preventing AD in ApoE4 non-carriers, while it is still possible that ACE inhibitor use may increase risk of developing AD [5].

The interaction between ApoE4 carrier status and ACE inhibitor use on AD (Fig. 1) may explain the previously reported conflicting findings of the relationship between ACE inhibitors and the risk of developing AD dementia [19], e.g., some studies showed a beneficial effect [5, 6], but another showed no effect or a harmful effect depending on the subclasses of ACE inhibitors [5]. Although the numbers were small, one clinical trial showed a beneficial effects on cognitive decline in AD [20], but other did not [21]. Since ApoE4 non-carriers and carriers may respond to ACE inhibitors differently, it is understandable that studies that do not control for differences in ApoE4 genotype may reach different conclusions. Another reason for the conflicting results among prior studies might be the failure to distinguish between central and peripheral ACE inhibitors since only peripheral ACE inhibitors are associated with an increased rate of AD development [5]. ACE inhibitors pass through the blood-brain barrier differently; peripheral inhibitors like enalapril cannot pass through the blood-brain barrier [22], while central inhibitors like lisinopril and trandolapril [23] can. Peripheral ACE inhibitors were more associated with a reduced risk of developing AD than central ACE inhibitors in our study (Table 2 and Fig. 2). ACE activity in blood serum is reported to be higher in the elderly who later developed AD than in those who did not [24].

ACE polymorphisms are reported to be associated with AD risk in some studies [25, 26]. Renin-angiotensin system (RAS) gene polymorphisms modify ACE inhibitors’ effect on cognitive function [27]. It is reported that the expression [28] and activity [29] of ACE are elevated in the AD brain and correlated with Braak stage [29]. All these studies suggest that ACE may be involved in AD pathogenesis [2] and may interact with the ApoE4 allele to influence this process. Genetically, some studies show that ApoE4 genotype interacts with the polymorphisms of ACE gene to increase the risk of developing AD [30, 31].

The mechanism of interaction of ApoE2 or ApoE3 and ACE inhibitors on delaying the dementia of AD (Table 2 and Fig. 2) is unclear. There are two possibilities. One possibility is that both ApoE4 and ACE inhibitor use may have a synergistic effect in reducing the clearance of Aβ[32, 33], a major component of AD pathology. Another possibility is that ACE inhibitors block ACE to generate angiotensin II, as abundant angiotensin II could cause cerebrovascular pathology to promote the AD pathology. Current studies in the literature argue that ACE inhibitors reducing the AD risk in the absence of ApoE4 is probably through decreasing angiotensin II and reducing cerebrovascular pathology rather than by affecting degrading Aβ. Angiotensin II infusion can induce cerebrovascular aneurysm and infarcts in ApoE −/− deficient mice [34], and talmisartan, an angiotensin receptor blocker (ARB), attenuates this effect [34]. Using a large clinical dataset, it has been shown that the combined use of ABR, which blocks the binding of angiotensin II to the receptor, and ACE inhibitors further reduced the number of incident AD and the progression of AD than ABR use alone [7, 35]. A pilot clinical trial did not find that a 4 month treatment with ramipril change the level of Aβ in cerebrospinal fluid [36]. Our previous study showed that in the presence of ApoE4, ACE inhibitor use was not associated with reduced ACE N-terminal activity, which is critical to produce angiotensin II [8]. Additionally, because another class of antihypertensive drugs, calcium channel blockers, are not associated with risk of AD [6, 7], we think that the probable effect of ACE inhibitors on AD is specific and not due to lowering blood pressure in itself.

Since ACE inhibitors are common antihypertensive medications used in the elderly, personalized medicine approaches may be important in AD intervention and prevention, especially among hypertensive patients for whom ACE inhibitors are considered. Our findings demonstrated that ACE inhibitors may delay the development of AD dementia in ApoE4 non-carriers, but have no such effect or some harmful effect when ApoE4 allele is present. However, our study was limited by the non-randomized nature of ACE inhibitor use and did not document the doses. As AD is a brain disease, central ACE inhibitors are expected to be more effective to delay the onset of AD than peripheral ACE inhibitors if a well controlled clinical trial is conducted. Nevertheless our study indicated the need to conduct a double-blind clinical trial to determine not only the preventive effect of ACE inhibitors on AD in the absence of ApoE4 but also a possible harmful effect of peripheral ACE inhibitors on the risk of AD in the presence of ApoE4.

## Figures and Tables

**Table 1:** Baseline demographic and medical status of non-ApoE4 and ApoE4 carriers in the NACC population

	ApoE4 − *n* = 3,338	ApoE4 + *n* = 1,492	DF	Chi square	*p* values
*Baseline Information*					
Age, year, mean + SD	77.3 ± 8.1	74.6 ± 7.1	1	–	<0.0001
School years, mean + SD	15.1 ± 3.2	15.2 ± 3.2	1	–	0.26
Caucasians, *n*/total (%)	2909/3338 (87%)	1227/1492 (82%)	1	20.2	<0.0001
Male, *n*/total (%)	1596/3338 (48%)	705/1492 (47%)	1	0.13	0.72
Current smoking, *n*/total (%)	145/3338 (4%)	39/1492 (3%)	2	8.5	0.01
Current alcohol abuse, *n*/total (%)	156/3338 (5%)	46/1492 (3%)	3	11.1	0.01
Follow-up time, year, mean + SD	3.5 ± 1.1	3.3 ± 1.2	1	–	<0.0001
MMSE, mean + SD	28.4 ± 1.9	27.9 ± 2.2	1	–	<0.0001
Amnestic MCI, *n*/total (%)	608/3338 (18%)	410/1492 (27%)	1	53.2	<0.0001
Non-amnestic MCI, *n*/total (%)	185/3338 (6%)	75/1492 (5%)	1	0.54	0.46
*Baseline Medical Conditions*					
Hypertension, *n*/total (%)	2677/3338 (82%)	1226/1492 (82%)	3	2.74	0.43
Diabetes, *n*/total (%)	637/3338 (19%)	259/1492 (17%)	3	11.0	0.01
History of stroke, *n*/total (%)	204/3165 (6%)	64/1464 (4%)	1	7.89	0.005
Heart failure, *n*/total (%)	227/3338 (7%)	61/1492 (4%)	3	14.3	0.003
*ACE Inhibitor Use*					
ACE inhibitor, *n*/total (%)	2237/3338 (67%)	1018/1492 (68%)	1	0.69	0.41
Central ACE inhibitor	1843/3338 (55%)	804/1492 (54%)	1	0.73	0.39
Peripheral ACE inhibitor	400/3338 (12%)	216/1492 (14%)	1	5.76	0.02
*Developed Alzheimer’s disease*					
Probable Alzheimer’s disease	457/3338 (14%)	443/1492 (30%)	1	174.12	<0.0001
Possible Alzheimer’s disease	275/3338 (8%)	156/1492 (10%)	1	6.23	0.01

Mean ± SD with *t* test or *n*/total (%) with chi square (χ^2^ test) are presented. *p* values for statistical significance are shown. MMSE, Mini-Mental State Examination; MCI, mild cognitive impairment; ACE, angiotensin converting enzyme.

**Table 2:** Effects of ApoE4 allele, ACE inhibitor use, and the interaction between ApoE4 status and ACE inhibitor use on Alzheimer’s disease

	Model I Alzheimer’s disease (*n* = 4,830)		Model II Alzheimer’s disease (*n* = 4,629)		Model III Alzheimer’s disease (*n* = 4629)	
	Odds Ratio (95% CI)	*p* value	Odds Ratio (95% CI)	*p* value	Odds Ratio (95% CI)	*p* value
ApoE4	2.40 (2.13, 2.72)	<0.0001	2.33 (2.04, 2.66)	<0.0001	1.46 (1.19, 1.78)	0.0003
Central ACEI	0.85 (0.74, 0.98)	0.03	0.79 (0.67, 0.93)	0.004	1.33 (1.02, 1.71)	0.03
Peripheral ACEI	0.68 (0.54, 0.86)	0.001	0.73 (0.57, 0.94)	0.02	1.55 (1.08, 2.23)	0.02
ApoE4*Central ACEI	–	–	–	–	0.44 (0.33, 0.60)	<0.0001
ApoE4*Peripheral ACEI	–	–	–	–	0.27 (0.16, 0.44)	<0.0001

Multivariate logistic analyses were used. ApoE4*ACE inhibitor, interaction between ApoE4 and ACE inhibitor (ACEI) use. Odds ratios with 95% confidence interval (95% CI) were shown for each variable in the models. We used each interval between annual visits as our analysis unit taking into account non-independence of study data due to repeated measures. *p* values for statistical significance are shown. Model I: Adjusting for age, gender, ethnicity, education, smoking, drinking and follow-up time. Model II: Model I plus diabetes, hypertension, stroke, heart failure, amnestic MCI and non-amnestic MCI. Model III: Model II plus the interaction between ApoE4 and central ACE inhibitors (ApoE4*Central ACEI) and the interaction between ApoE4 and peripheral ACE inhibitors (ApoE4*Peripheral ACEI).
